# Robust Vehicle Detection and Counting Algorithm Adapted to Complex Traffic Environments with Sudden Illumination Changes and Shadows

**DOI:** 10.3390/s20092686

**Published:** 2020-05-08

**Authors:** Yue Chen, Wusheng Hu

**Affiliations:** School of Transportation, Southeast University, Nanjing 210096, China; cy_cheny_cy@163.com

**Keywords:** real-time background, vehicle detection, vehicle counting, Normative-Lane and Non-Normative-Lane

## Abstract

The real-time vehicle detection and counting plays a crucial role in traffic control. To collect traffic information continuously, the access to information from traffic video shows great importance and huge advantages compared with traditional technologies. However, most current algorithms are not adapted to the effects of undesirable environments, such as sudden changes in illumination, vehicle shadows, and complex urban traffic conditions, etc. To address these problems, a new vehicle detection and counting method was proposed in this paper. Based on a real-time background model, the problem of sudden illumination changes could be solved, while the vehicle shadows could be removed using a detection method based on motion. The vehicle counting was built on two types of ROIs—called Normative-Lane and Non-Normative-Lane—which could adapt to the complex urban traffic conditions, especially for non-normative driving. Results have shown that the methodology we proposed is able to count vehicles with 99.93% accuracy under the undesirable environments mentioned above. At the same time, the setting of the Normative-Lane and the Non-Normative-Lane can realize the detection of non-normative driving, and it is of great significance to improve the counting accuracy.

## 1. Introduction

With the development of Intelligent Transport Systems (ITS), real-time traffic monitoring has become one of the most important technologies. There are various sensors used to collect traffic information, which could be categorized into two types: hardware-based sensors and software-based sensors [1]. The former one is a kind of traditional sensor for information collection, which is based on a dedicated equipment, such as infrared sensor, electromagnetic induction loop coil, ultrasonic sensor, radar detector, piezoelectric sensor and so on, while the latter is based on the complex image-processing techniques over surveillance video cameras most. For the hardware-based sensors, the loop coil and the piezoelectric sensor need to be located within the pavement, which will cause huge damage to the road, and the installation and irregular maintenance require an interruption in traffic; the infrared sensor, the ultrasonic sensor and the radar detector will lose their functionalities when affected by environmental factors [2], such as signal interference. Comparing with traditional sensors, a video camera shows a huge advantage with its flexibility and low cost. More importantly, with a high video frame rate (more than 24 frames per second), the traffic information collected through video is more real-time and the sampling rate is much higher than traditional sensors. Therefore, there has been a trend to capture vehicle information through surveillance video cameras in recent years.

For most algorithms, vehicle detection is the first step before vehicle counting. The techniques for vehicle detection can be categorized into feature-based methods and motion-based methods. The former is based on the visual features [3], such as color [4], texture [5], edge [6], contour [7], symmetry [8], or the characteristic parts of the vehicle, such as vehicle lights [9], license plates [10], windshield [11], etc. Multi-feature methods are most commonly used in practice. In [12], a hierarchical generative model was built to recognize compositional object categories by large intra-category variance. The extraction of low-level features is fast and convenient, but it cannot represent all of the useful information efficiently. To solve this problem, a machine-learning method [13] was brought into vehicle detection. For example, a deep neural network based on visual features was trained in [14], but its drawback of time-consuming makes it unable to meet the real-time requirements.

Unlike the feature-based method, the motion-based method is faster, for it does not involve any prior knowledge and parameters. The interframe differencing method and the background subtraction method are two of the most characteristic examples. Interframe differencing was widely used in the early days [15], but it cannot be adapted to the detection of slow-moving targets and suffers from the holes seriously [16]. Background subtraction is performed based on a binary image generated by the difference between moving targets and static background [1], and it is also effective against slow-moving targets. However, this method is susceptible to environment changes in complicated circumstances, such as sudden illumination changes on a cloudy day. Moreover, the shadow of the vehicle may induce shape distortions and object fusions [17], which will affect the results of vehicle detection and make the problems more complicated [18], and there is not a robust enough solution to deal with the shadow of vehicles so far.

For counting steps, vehicle tracking and extracting information using the Regions of Interests (ROIs) are two of the most mature methods. The former one achieves the goal of vehicle counting by tracking the same vehicle in a series of frames, and the use of the Kalman filter [19] or exploiting feature tracking [20] were the most common approaches. However, it does not suit online operation with its computational complexity. In this case, a faster method based on the ROIs was proposed, which has little computational cost. In [21], a lineal ROI—a kind of virtual detection line—set on each lane of the road was used to count vehicles, while double virtual lines [22] and small virtual loops [23] could also be applied. However, the existing algorithms can only obtain a relatively accurate result under ideal conditions. When faced with undesirable environments, such as sudden illumination changes and vehicle shadow, or on a complex urban road rather than a simple highway, the mistakes of misidentifying or missing a vehicle will be extremely serious.

In this paper, a new vehicle detection and counting method will be proposed. Combining with a real-time background model, a motion-based algorithm for removing vehicle shadows and an optimization of image by filling holes and denoising, the adverse effects of sudden illumination changes and vehicle shadows can be well overcome. For vehicle counting, two types of ROIs—called Normative-Lane and Non-Normative-Lane—will be innovatively introduced into our system, which can detect non-normative driving efficiently. At the same time, the driving state of a vehicle can also be recorded, which greatly improves the precision of vehicle counting. Table 1 summarizes the characteristics and advantages of our work compared with existing state-of-the-art methods.

## 2. Methodology

**Figure 1:**
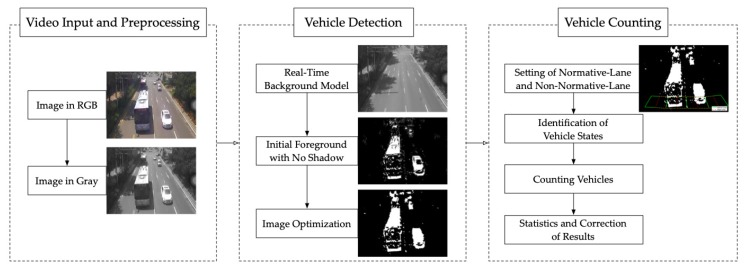
Block diagram of the proposed framework.

Our methodology is based on the surveillance traffic video, and it contains two main parts: vehicle detection and vehicle counting, which will be described in the following sections. The block diagram of the proposed framework is shown in Figure 1 (the sequences in Figure 1 are from Video Mofan Rd [28], which will be introduced in detail in Section 2.1).

### 2.1. Description of Datasets

To validate the performance of our proposed system under the undesirable environments mentioned above, four traffic videos were selected for experiments. The first two are benchmark datasets on the highway, while the other two were recorded by ourselves in Nanjing, Jiangsu, China, which are on an expressway and an urban road, respectively. The first video is called Highway, which can be obtained from the Change Detection Benchmark [29]; the second one is called M-30-HD, which can be obtained from the Road-Traffic Monitoring (GRAM-RTM) dataset [30]; the third and fourth ones are called Mofan Rd and Zhongshan Rd, which can be obtained from [28]. The pixel ratio of all these videos was adjusted to a uniform pixel ratio (4:3) by cutting and compressing, which could reduce the running time of the computer and unify the video processing, and the frame rate of all these videos is 30 fps. The attributes of the datasets are shown in Table 2.

As described in Section 1, there are two main challenges in the detection part. The first is the effect of sudden illumination changes on the establishment of background, such as the cloud occlusion and dissipation on cloudy days. The second one is how to remove the shadow of moving vehicles on sunny days, especially when the sun is tilting. Moreover, the traffic conditions also matter a lot. There are three kinds of adverse effects, mainly: high proportion of large vehicles, high traffic density or traffic flow, and serious non-normative driving. These adverse effects may be less common on highways but have a greater impact on urban roads. Also, the poor camera shooting angle can have a big impact on the accuracy of counting, especially for tall and large vehicles. Therefore, we selected these four videos containing the above challenges to verify that our proposed method offers a robust vehicle detection and counting system.

Video Highway contains a poor camera shooting angle and a few vehicle shadows, and the waving trees in this scene may have some bad effects; Video M-30-HD mainly contains sudden illumination changes. However, the detection environments of these two benchmark datasets on the highway are relatively ideal. For example, there are no large amounts of vehicle shadow and complex traffic conditions, which are often faced on urban roads, such as high proportion of large vehicles (especially buses), high traffic density or flow and serious non-normative driving.

In order to further verify the effectiveness and robustness of our method, two more challenging videos recorded by ourselves were chosen for testing, where sudden illumination changes and poor camera shooting angle are included in Zhongshan Rd, while a large amount of vehicle shadow and high proportion of large vehicles are included in Mofan Rd. Moreover, the challenges of high traffic density and serious non-normative driving happened in both of these two videos.

**Figure 2:**
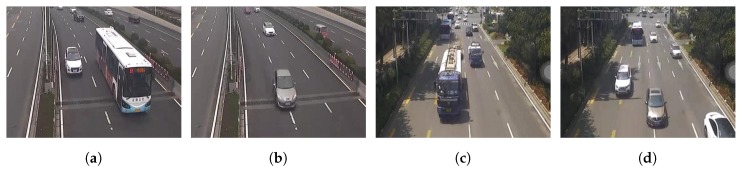
The instruction of Zhongshan Rd and Mofan Rd.

The weather of Zhongshan Rd is cloudy, which will bring the challenge of sudden illumination changes. Figure 2a,b shows that the shooting angle is relatively tilted, which will make the tall and large vehicles be easily detected repeatedly in two lanes, while the non-normative driving is also serious, just like Figure 2b shows. For Mofan Rd, Figure 2c,d shows the serious vehicle shadows clearly and the proportion of large vehicles (especially buses) is really high, and the non-normative driving is more serious in this urban road. Coupled with high traffic density, all of these adverse factors increase the difficulty of detection and counting.

It is also worth noting that we have chosen enough video frames (54,000) for more cases to validate our methods. The characteristics and challenges of these roads are shown in Table 3.

### 2.2. Detection Based on Motion

Getting an accurate detection result is an essential process before the counting part. For ideal scenes, this goal is easy to achieve. However, when the detection environment is poor, many algorithms become ineffective. For example, the sudden change of illumination will bring great challenges to the establishment of real-time background, which may affect the extraction of real foreground directly; the shadow moving with vehicle caused by the sunlight could be easily mistaken for the foreground, which greatly increases the chances of unwanted counting; at the same time, incomplete foreground extraction may cause an error of missing the target vehicles.

In this section, a vehicle detection algorithm based on motion was proposed. Our algorithm could be divided into four steps. A real-time background model should be set at first, which can resist sudden illumination changes in cloudy weather or other situations. Second, an algorithm for removing vehicle shadows based on motion was proposed, which can greatly improve the accuracy of foreground extraction. Then, a vehicle filling method based on vehicle edge was studied in this paper, which could be used as a supplementary means for extracting a more complete foreground. At last, denoising methods were brought into our system to obtain an optimized foreground extraction.

#### 2.2.1. Real-Time Background Model

(i) Initial Background

A background of a scene only consists of static pixels, and it is the basis for foreground extraction. However, for a highway or an urban road, it is extremely rare that there is no moving object in a frame, and even if this particular frame exists, it is hard to be found. Therefore, to establish a background model, the general method is analyzing the distribution characteristics of pixel value at each pixel position in a series of original frames, and selecting or calculating the static pixel values. Among the existing mature methods, there are three main approaches: the Gaussian mixture model (GMM) [31], the statistical median model (SMM) [32], and the multi-frame average model (MAM) [33].

In terms of extraction accuracy, the GMM is better than the SMM and the MAM, while the SMM is better than the MAM. However, the GMM cost 40–50 times longer than the other two, which makes it unsuitable for online video. In addition, the GMM contains two parameters: α (the learning constant) and *N* (the number of video frame should be analyzed in the model), while the other two only contain *N* [34]. Considering the accuracy and the running speed comprehensively, the SMM is the most suitable for online background extraction among these three methods.

Let height and width represent the height and the width of an image, and each pixel position could be expressed as (i,j), where i=1,2,⋯,height, j=1,2,⋯,width. The total number of frames is represented by duration, and the sequence number of each frame could be expressed as *k*, where k=1,2,⋯,duration. The first step is changing RGB images into gray images. We call it $I_{k}^{gray}$ here, which represents the gray value vector of pixels for Frame *k*.

For SMM, there is only one parameter (*N*) that needs to be tuned. We set N=30 here, and these *N* frames were selected per second. For a video with a frame update rate of 30 fps, this algorithm only needs to be operated every 30 s, which greatly reduces the computer running time. The initial background extraction at each position could be expressed as:

(1) $${BG\_initial}_{n}^{gray}=\underset{k}{median}I_{k}^{gray}$$

(2) k=N∗rate∗(n−1)+1,rate∗1+[N∗rate∗(n−1)+1],⋯,rate∗(N−1)+[N∗rate∗(n−1)+1]

where rate is the frame rate of video, *n* represents the sequence number of the initial background, and ${BG\_initial}_{n}^{gray}$ is the gray value vector of the $n^{th}$ initial background.

(ii) Real-Time Background

**Figure 3:**
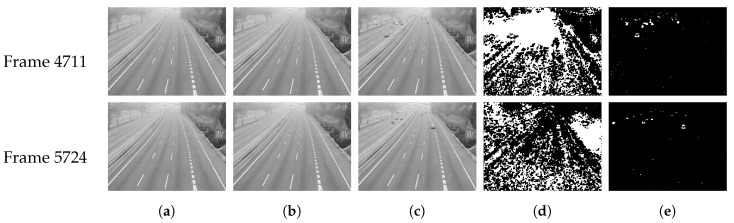
Comparison results of initial background and real-time background. (**a**) The initial background. (**b**) The real-time background. (**c**) The current frame. (**d**) The extraction of the foreground using initial background. (**e**) The extraction of the foreground using real-time background.

A good background must adapt to the gradual or sudden illumination changes [35], such as the changing time of a day or clouds, etc. However, the initial background has poor resistance to such situations, especially for sudden changes. To illustrate this phenomenon better, we chose the Video M-30-HD [30] with lots of sudden illumination changes, which was shot on a cloudy day. Two frames were selected for display here, which were badly affected by the cloudy weather. The selected frame numbers (*k*) are 4711 and 5724 and the result of foreground extraction using the initial background is shown in Figure 3c.

It is clear that not only were the actual foreground pixels extracted, but a large part of the background was also mistaken for the foreground. This is because the establishment of initial background must be based on a series of images, which results that the difference between the initial background and the real-time background is always present. Worse, it will not be effective even if the running frequency of the algorithm is greatly increased at the cost of high computational complexity. In this case, the solution is modifying the background of each frame in real time based on the initial or previous background. Toyama [36] proposed an adaptive filter based on the previous background in 1999:

(3) $$BG_{k}^{gray}=\left(1-\alpha \right)BG_{k-1}^{gray}+\alpha I_{k}^{gray}$$

where $BG_{k}^{gray}$ is the gray value vector of the current background (Frame *k*), and $BG_{k-1}^{gray}$ is the previous background (Frame k−1); α is the parameter that decides the rate of adaptation in the range 0–1. However, α is an experienced parameter, which is hard to be tuned appropriately for different cases. Moreover, there will be a cumulative error if the previous background is extracted inaccurately. In this case, we proposed an adaptive algorithm to obtain a real-time background, which only contains one parameter that is easily determined.

**Figure 4:**
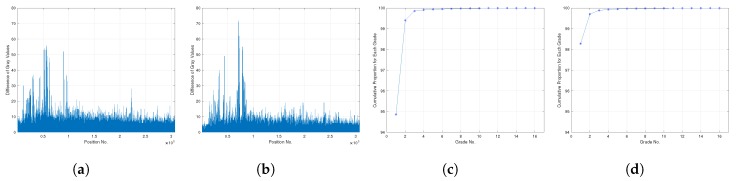
Square distribution of the difference between initial background and current frame and relevant cumulative distribution. (**a**,**b**) The difference distribution of gray values for each selected frames. (**c**,**d**) The cumulative distribution for each grade.

First of all, the difference of gray value between the initial background and the current frame should be studied, which could be expressed as:

(4) $$\delta_{k}^{gray}=\left|I_{k}^{gray}-{BG\_initial}^{gray}\right|$$

where $\delta_{k}^{gray}$ is the difference vector of gray values between the initial background and current frame, and the $\delta^{gray}$ of two frames are shown in Figure 4a,b. It is easy to see that $\delta^{gray}$ could be divided into three classes at each position, that is:

(5) $$Class_{k}\left(i,j\right)=\left\{\begin{array}{cc} 1, & \delta_{k}^{\mathrm{gray}}\left(i,j\right)=0\\ 2, & \delta_{k}^{\mathrm{gray}}\left(i,j\right)\in \left(0,T^{\mathrm{delta}}\right]\\ 3, & \delta_{k}^{\mathrm{gray}}\left(i,j\right)\in \left(T^{\mathrm{delta}},255\right] \end{array}\right.$$

where $T^{delta}$ is the threshold of the classification that needs to be tuned.

Class 1 means the position (i,j) for Frame *k* belongs to the background, Class 3 means (i,j) belongs to the foreground, while Class 2 also means (i,j) belongs to the background but the initial background needs to be adjusted with $I^{gray}$.

In order to determine the value of $T^{delta}$, the distribution of $\delta^{gray}$ should be studied more clearly. It is easy to find that $\delta^{gray}$ fluctuates directly between 0–80, so we divided 100 into 20 grades with an interval of five to calculate the percentage of position numbers, and the cumulative distribution of each grade is shown in Figure 4c,d. It is obvious that more than 90% of $\delta^{gray}$ are distributed in [0,5], more than 99% are distributed in [0,10], and more than 99.5% are distributed in [0,15]. Therefore, for the sake of careful estimation, we could conclude that $T^{delta}$ should be set in [5,15].

Based on the study above, we proposed an adaptive algorithm to obtain the real-time background, which could be expressed as:

(6) $$BG_{k}^{gray}=\left\{\begin{array}{cc} {BG\_initial}^{gray}, & \delta_{k}^{\mathrm{gray}}\in 0\cup \left(T^{\mathrm{delta}},255\right]\\ w^{I}*I_{k}^{gray}+w^{BG}*{BG\_initial}^{gray}, & \delta_{k}^{\mathrm{gray}}\in \left(0,T^{\mathrm{delta}}\right] \end{array}\right.$$

(7) $$w^{I}=\frac{\delta_{k}^{gray}}{T^{delta}},\;w^{BG}=1-\frac{\delta_{k}^{gray}}{T^{delta}}$$

where $w^{I}$ is the weight of the current frame and $w^{BG}$ is the weight of the initial background.

According to Formula (6), the higher the $T^{delta}$, the lower the $w^{I}$. In this case, $w^{I}$ and $w^{BG}$ can make up for the errors of setting $T^{delta}$ adaptively together. Therefore, the parameter $T^{delta}$ has no vital effect on the generation of real-time background, which greatly improves the adaptability and accuracy of the algorithm. As mentioned before, we set $T^{delta}$ in [5,15] here, and the extraction of foreground using our real-time background is shown in Figure 3e. As can be seen, the real-time backgrounds overcome the adverse effects of sudden illumination changes well.

#### 2.2.2. Initial Foreground with No Shadow

(i) Light and Dark Foreground

Subtracting the current frame from the background directly and converting it into a binary image is the most used method to extract the foreground, however, it will remain the shadow unwanted. As we all know, shadows have three features [37] different from moving vehicles, which are intensity values, geometrical properties, and light directions. Based on the features, the shadow will be darker than the background, and it has nothing to do with the pixel value of the vehicle that produces it. In this case, the foreground image could be divided into two parts for analysis:

(8) $${FG\_dark}^{gray}=\left\{\begin{array}{cc} BG^{gray}-I^{gray}, & \mathrm{if}\;{\mathrm{BG}}^{\mathrm{gray}}\geq I^{\mathrm{gray}}\\ 0, & \mathrm{otherwise} \end{array}\right.$$

(9) $${FG\_light}^{gray}=\left\{\begin{array}{cc} I^{gray}-BG^{gray}, & \mathrm{if}\;{\mathrm{BG}}^{\mathrm{gray}}<I^{\mathrm{gray}}\\ 0, & \mathrm{otherwise} \end{array}\right.$$

where ${FG\_dark}^{gray}$ is the difference vector of gray value between current frame and background, which only contains the darker pixels, and ${FG\_light}^{gray}$ only contains the lighter pixels. Moreover, the Otsu’s method [38] was used here to get ${FG\_dark}^{binary}$ and ${FG\_light}^{binary}$.

**Figure 5:**
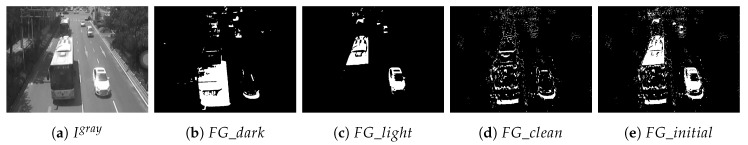
The extraction of the initial foreground on a sunny day.

The Video Mofan Rd [28] on an urban road was chosen as an example, which was recorded by ourselves on a sunny day and contains lots of vehicle shadows. We selected a frame (k=29,825) which contains a large bus and some small vehicles for display. The FG_dark and FG_light are shown in Figure 5b,c. As we can see, the FG_light contains no shadow. Although the vehicles obtained in this way were incomplete, to get ‘clean’ vehicles, we only choose the FG_light as a part of initial foreground and abandon the FG_dark for the time being.

(ii) Removing Shadows

Most algorithms for removing shadows are based on color features and run in a color model. The Hue-Saturation-Intensity (HSI) model could be used to detect the shadows, which is based on the fact that the chromaticity information will not be affected by the change of lighting. By selecting a region which is darker than its neighboring regions but has similar chromaticity information, the shadow could be detected. Cucchiara et al. [39] proposed an algorithm to achieve this goal:

(10) $$shadow=\left\{\begin{array}{cc} true, & \mathrm{if}\left(T_{1}^{I}\leq \frac{I^{I}}{{\mathrm{BG}}^{I}}\leq T_{2}^{I}\right)\wedge \left(\right|I^{S}-{\mathrm{BG}}^{S}|\leq T^{S})\wedge (|I^{H}-{\mathrm{BG}}^{H}\left|\leq T^{H}\right)\\ false, & \mathrm{otherwise} \end{array}\right.$$

where shadow is the judgement of shadow; $I^{I}$, $I^{S}$, $I^{H}$ are the color vectors of the current frame in the HSI model, while $BG^{I}$, $BG^{S}$, $BG^{H}$ are the color vectors of background; $T_{1}^{I}$, $T_{2}^{I}$, $T^{S}$ and $T^{H}$ are the four parameters to be determined.

There are four parameters to be determined in this algorithm, which greatly increases the instability of the judgement results. Even in a similar scene, the results will vary greatly under different lighting levels (such as different times of one day), which means the parameters need to be adjusted constantly. More importantly, this algorithm will also eliminate parts of the vehicle when the color of the vehicle itself is similar to the shadow color, which leads to the absence of vehicle information. Moreover, an algorithm based on a color model needs longer running time than the gray model.

Since such parameters are difficult to determine, we proposed a shadow removal method without parameters. As mentioned above, the chromaticity of shadow is not affected by the change of illumination for some cases. Based on it, we could suppose that the shadow of a vehicle will remain the same in a very short interval, such as an interval between two frames. Therefore, a pixel position can be judged as shadow if its value remains the same among the current frame and two adjacent frames. In this case, a frame with the shadow removed can be expressed as:

(11) $${FG\_clean}^{binary}=\left\{\begin{array}{cc} 0, & \mathrm{if}\;\left(I_{k}^{\mathrm{gray}}=I_{k-1}^{\mathrm{gray}}\right)\wedge \left(I_{k}^{\mathrm{gray}}=I_{k+1}^{\mathrm{gray}}\right)\\ 1, & \mathrm{otherwise} \end{array}\right.$$

where ${FG\_clean}^{binary}$ is the image with shadow removed. As we can see in Figure 5d, this algorithm works well in removing shadow, but it may cause some holes in the vehicles.

(iii) Initial Foreground

Combining the result of the previous two steps, the initial foreground can be expressed as:

(12) $${FG\_initial}^{binary}={FG\_light}^{binary}\cup {FG\_clean}^{binary}$$

As shown in Figure 5e, the shadow has been removed successfully but the vehicles are somewhat incomplete, and more noise was brought. Therefore, some methods for fulfilling the vehicles and denoising are necessary and they will be described in the next sections.

For a scene without shadow, it is not necessary to operate the algorithm for removing shadow. After all, this algorithm may make the vehicle incomplete and bring some noise. As mentioned above, the reason for abandoning FG_dark is that the darker foreground contains vehicle shadows. Therefore, the FG_light and FG_dark should be both included as initial foreground for scenes with no shadow. In this case, the initial foreground for a scene with no shadow could be expressed as:

(13) $${FG\_initial}^{binary}={FG\_light}^{binary}\cup {FG\_dark}^{binary}$$

**Figure 6:**
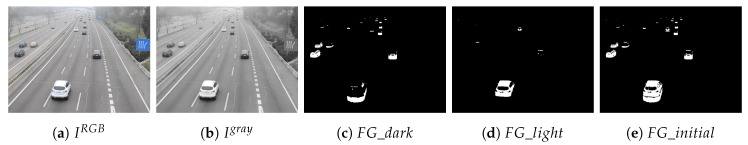
The extraction of the initial foreground on a cloudy day.

A frame (k=1169) from Video M-30-HD [30] was selected for display, shown in Figure 6.

#### 2.2.3. Image Optimization

After the foreground extraction based on motion is completed, it is necessary to fill vehicle holes and denoise to obtain a better foreground.

(i) Filling Image with Edge

Let’s review the shadow removal algorithm in Section 2.2.2. According to Formula (11), the binary image FG_clean is obtained by taking the relative complementary sets of adjacent frames ($I_{k-1}^{gray}$ and $I_{k+1}^{gray}$) in the current frame ($I_{k}^{gray}$), and this is also the reason why there are more holes in FG_clean. In this case, it is necessary to analyze the relationships between the current frame and two adjacent frames again separately from the perspective of the gray value. Let’s analyze the difference in the gray model, which could be expressed as:

(14) $${I\_dif}_{k}^{gray}=\left\{\begin{array}{cc} 0, & \mathrm{if}\;I_{k}^{\mathrm{gray}}=I_{k-1}^{\mathrm{gray}}\\ I_{k}^{gray}, & \mathrm{otherwise} \end{array}\right.,\;{I^{\prime }\_dif}_{k}^{gray}=\left\{\begin{array}{cc} 0, & \mathrm{if}\;I_{k}^{\mathrm{gray}}=I_{k+1}^{\mathrm{gray}}\\ I_{k}^{gray}, & \mathrm{otherwise} \end{array}\right.$$

where ${I\_dif}_{k}^{gray}$ is the difference gray image between current frame and previous frame, while ${I^{\prime }\_dif}_{k}^{gray}$ is the difference between the current frame and next frame.

Through a further analysis, we found that the difference gray image may contain noise, but the edge of them contains almost nothing more than the actual vehicle profiles. At this point, the edges of ${I\_dif}_{k}^{gray}$ and ${I^{\prime }\_dif}_{k}^{gray}$ play an important role in filling the holes. The edge was extracted with canny method [40] in this paper, which could be expressed as:

(15) $${FG\_edge}^{binary}=edge\left\{{I\_dif}_{k}^{gray}\right\}\cup edge\left\{{I^{\prime }\_dif}_{k}^{gray}\right\}$$

where edge{∗} is the edge extraction algorithm. $edge\{{I\_dif}_{k}^{gray}\}$ and $edge\{{I^{\prime }\_dif}_{k}^{gray}\}$ is the edge of ${I\_dif}_{k}^{gray}$ and ${I^{\prime }\_dif}_{k}^{gray}$, while ${FG\_edge}^{binary}$ is the extracted edge of current frame.

For a scene with no shadow, we found that the edge obtained from FG_light was cleaner, while the edge of FG_dark may contain more noise. Therefore, the extraction of the edge should based on FG_light only, which could be expressed as:

(16) $${FG\_edge}^{binary}=edge\left\{{FG\_light}^{binary}\right\}$$

**Figure 7:**
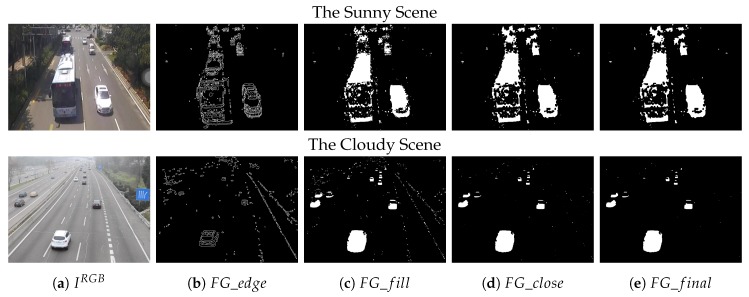
The extraction of the optimized foreground.

After the edge has been extracted, the holes in vehicle could be filled with edge. The filling method proposed by Pierre Soille [41] works well, which could be expressed as:

(17) $${FG\_fill}^{binary}=fill\left\{{FG\_initial}^{binary}\cup {FG\_edge}^{binary}\right\}$$

where fill{∗} is the filling algorithm, and ${FG\_fill}^{binary}$ is the filled image. It is worth noting that a simple median filtering operation [42] on the FG_fill is very effective in removing redundant noise caused by the edge. The filling and filtering results are shown in Figure 7c.

(ii) Morphological Closing

As shown in Figure 7c, the FG_fill still contains some big holes and some extra noise. At this point, it becomes necessary to perform a closing operation [43] on FG_fill:

(18) $${FG\_close}^{binary}=close\left\{{FG\_fill}^{binary}\right\}$$

A median filtering operation could be done again and the final foreground is shown in Figure 7e.

According to Figure 7, the algorithm works well on a cloudy day. As for a shadow scene, there are almost no vehicle shadows left in FG_final and the algorithm works better for small vehicles. Although there are still some holes in large buses, it does not affect the vehicle counting discussed in Section 2.3.

### 2.3. Vehicle Counting

At present, the method of setting ROIs is often adopted to realize the counting of vehicles. However, the problems of missing vehicles or detecting vehicles redundantly often occur by setting a detection line or a detection area simply. Worse, this simple setting will miss vehicles that do not drive following the normative lane, which means that the vehicles drive on the traffic index line, and such non-normative driving is very common on complex urban roads.

Different from the previous algorithms, we will propose a vehicle counting method based on lane division to avoid the errors of redundant or missed vehicle detecting. Moreover, the algorithm can realize the detection of vehicles driving on the traffic index line.

#### 2.3.1. Setting of Normative-Lane and Non-Normative-Lane

**Figure 8:**
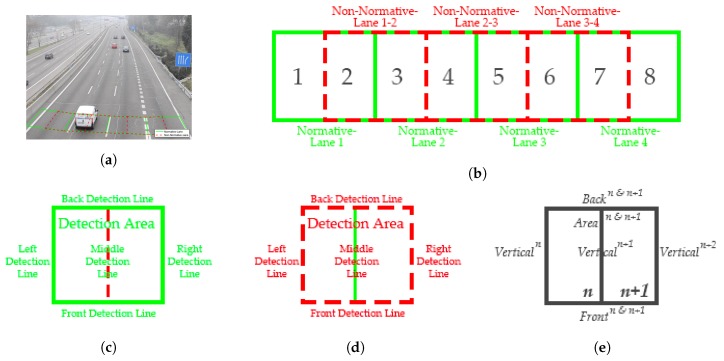
The instruction of Regions of Interests (ROI). (**a**) The setting of the ROIs. (**b**) The ROIs for a four-lane road. (**c**) The Normative-Lane ROI. (**d**) The Non-Normative-Lane ROI. (**e**) The normalized ROI.

The lane was divided into two categories in this paper: Normative-Lane and Non-Normative-Lane. The Normative-Lane is a kind of lane that vehicles should drive on following the traffic rules, while the Non-Normative-Lane is a kind of non-normative lane and not in accordance with the traffic laws, that is, it contains traffic index line. The corresponding ROIs of these lanes would be set up respectively, as shown in Figure 8a,b. Unlike previous algorithms, the ROI settings combine both detection line and detection area. More importantly, not only the horizontal detection line was brought, the vertical detection line was also brought into our system to increase the accuracy of vehicle counting.

The ROI should be positioned directly below the camera as far as possible to avoid additional errors due to the tilt of vision field. For the Normative-Lane ROI, we set up the width according to the width of lanes; for the Non-Normative-Lane ROI, we set it in the middle of two Normative-Lanes and with the same width. In this way, there exits an overlap between the Normative-Lane ROI and the Non-Normative-Lane ROI. Moreover, what needs to be explained is that the ‘adjacent lane’ in this paper refers to the Normative-Lane and the Non-Normative-Lane, instead of the regular sense of an adjacent lane.

As for the length of ROI, we set it less than the safe vehicle spacing. The spacing is generally divided into two parts: the reaction distance and the braking distance. According to [44], the reaction distance can be expressed as:

(19) $$d_{r}=St$$

where $d_{r}$ is the reaction distance, *S* is the initial speed of vehicle and *t* is the reaction time. Involving braking reactions, the American Association of State Highway and Transportation Officials (AASHTO) mandated the use of 2.5 s as *t* for most computations [45]. In this case, a driver driving at a very low speed, such as 10 km/h, will need at least 7 m for safety. Therefore, we set the length to be less than 7 m, which can ensure that there is at most one vehicle in a ROI and avoid missing vehicles on the same lane. Both the Normative-Lane ROI and the Non-Normative-Lane ROI are set to the same length. Moreover, the ROIs should be fine-tuned according to the tilt angle of the video, as shown in Figure 8a.

In this case, there will be seven origins for a four-lane road. For a Normative-Lane ROI or a Non-Normative-Lane ROI, a detection area and five kinds of detection lines are included, which are front and back detection line, middle detection line, and left and right detection line, shown in Figure 8c,d. Therefore, what we need to record is the proportion of moving vehicles occupied in the detection area and the five kinds of detection lines in each foreground binary image.

It is worth noting that the left or right detection line of two adjacent lanes—as explained above, the adjacent lanes refer to the Normative-Lane and the Non-Normative-Lane—coincides with the middle detection line, and the front and back detection lines of them overlap. For the convenience of research, we normalized these two ROIs. Shown in Figure 8b, we took the middle detection line of seven lanes as the boundary and divided the whole detection region into eight small regions first. Then we combined the adjacent small regions in pairs to form a normalized ROI, called ${ROI}^{n\&n+1}$, where *n* and n+1 represent the $n^{th}$ and ${n+1}^{th}$ small region. For a normalized ROI, the detection area, the front and back detection line can be called Area, Front, Back, while the middle, left and right detection line can be represented by three Vertical lines, respectively, as shown in Figure 8e and Table 4.

#### 2.3.2. Identification of Vehicle States

(i) Nodes and States of Vehicles

Before counting vehicles, we need to identify and record the state of vehicle in each ROI. For a detected vehicle, a complete detection process can be divided into seven stages with four states and three nodes. These four states are:

1. State 0: Out of the ROI (the vehicle has not entered the ROI);
2. State 1: In the ROI (the vehicle has entered the ROI and occupied in the front detection line, but out of the back detection line);
3. State 2: Still in the ROI (the vehicle has left the front detection line and occupied in the back detection line);
4. State 0’: Out of the ROI (the vehicle has left the ROI).

**Figure 9:**
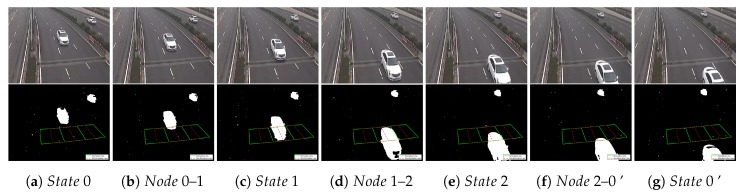
The instruction of nodes and states. (The sequences are from Video Zhongshan Rd [28]).

Correspondingly, the three nodes are: Node 0–1, Node 1–2 and Node 2–0’. A complete detection process with seven stages is shown in Figure 9.

(ii) Identification of States

(a) Restrictions of Parameter

The identification of these three important states for Frame *k* is realized through six parameters of the proportion that vehicles occupied in the detection area lines. As shown in Figure 8 and Table 4, the study of these six parameters can be turned into the study of four parameters, that is, $Area_{k}$, $Front_{k}$, $Back_{k}$, and $Vertical_{k}$.

The occupied proportion of detection area and middle detection line are two of the most obvious criterions, which should be at a low value in State 0 and at a high value in State 1 or State 2, while the front and back detection line are the keys to distinguishing between State 1 and State 2. The left and right detection line is a guarantee for vehicles driving in this ROI. Detailed restrictions are shown in Table 5. There are six thresholds corresponding to the six parameters, which are $T^{Area}$, $T^{Mid}$, $T^{Front}$, $T^{Back}$, $T^{Left}$ and $T^{Right}$. In general, the $T^{Front}$ and $T^{Back}$ can be set to the same value, and so can the $T^{Left}$ and $T^{Right}$. With the strict restrictions of parameter, some dynamic objects that are not vehicles can be removed, such as electric bicycles and the noise caused by environmental mutations.

(b) Restrictions of State

It is not accurate to judge the state of a vehicle only by the six occupied proportions, but the judgements of the states of its front and adjacent vehicles are also necessary.

As mentioned above, the length of the ROI is less than the vehicle spacing. Therefore, only when the front vehicle has completely left, that is, when the ROI state is in State 0, can a new-coming vehicle be detected. For adjacent lanes, it is easy to know that it is impossible for two adjacent vehicles to be in State 1 at the same time because there is an overlap between Normative-Lane and Non-Normative-Lane (Figure 8b). In this case, only when the ROI states of adjacent lanes are in State 0 or State 2 can a vehicle be detected. The detailed restrictions are shown in Table 5. Through the restrictions of state, multiple detection of its front and adjacent vehicles can be effectively avoided. Meanwhile, non-normative driving behaviors can be detected effectively.

#### 2.3.3. Counting Vehicles

Through the detection in the previous section, a moving object could be detected successfully. Whether it belongs to a vehicle, however, requires a further identification. In this section, we will introduce how to filter and classify the detected objects with State 1 and State 2.

The duration of State 1 represents the frames taken to pass through the front detection line for a vehicle, while the duration of State1&2 represents the frames taken to pass through the ROI. In this case, the frames occupied by the detected vehicle in State 1 and State1&2 could be used to further filter the detection results. Meanwhile, the duration of State 1 and State 2 can also be used to qualitatively determine the size of a vehicle on a road with a relatively uniform speed, such as on a highway or on an urban expressway.

**Figure 10:**
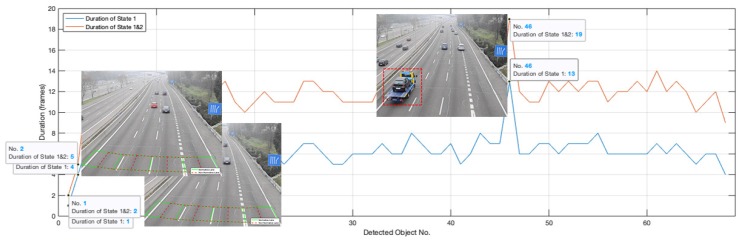
The interpretation for the meaning of State 1 and State1&2.

We selected State 1 and State 2 of Normative-Lane 1 in Video M-30-HD [30] for analysis. As shown in Figure 10, the State 1 duration of the first detection is one frame, while the duration of State1&2 is two, which clearly indicates that the first detection belongs to noise rather than a vehicle. The same is true for the second detection. Therefore, these two detected objects should be identified as noise. Moreover, the State 1 and State1&2 duration of the 46th detection are much longer than the other detected objects, which clearly indicates that the 46th detection is in a larger size. The detected object can be expressed as:

(20) $${Detected}_{n}=\left\{\begin{array}{cc} noise, & \mathrm{if}\;\left(0<{\mathrm{State}\;1}_{n}<T_{1}^{\mathrm{State}\;1}\right)\wedge \left(0<{\mathrm{State}\;1\&2}_{n}<T_{1}^{\mathrm{State}\;1\&2}\right)\\ Small, & \mathrm{if}\;\left(T_{1}^{\mathrm{State}\;1}<{\mathrm{State}\;1}_{n}<T_{2}^{\mathrm{State}\;1}\right)\wedge \left(T_{1}^{\mathrm{State}\;1\&2}<{\mathrm{State}\;1\&2}_{n}<T_{2}^{\mathrm{State}\;1\&2}\right)\\ Large, & \mathrm{if}\;\left(T_{2}^{\mathrm{State}\;1}<{\mathrm{State}\;1}_{n}\right)\wedge \left(T_{2}^{\mathrm{State}\;1\&2}<{\mathrm{State}\;1\&2}_{n}\right) \end{array}\right.$$

where ${Detected}_{n}$ is the $n^{th}$ detection, ${State\;1}_{n}$ and ${State\;1\&2}_{n}$ are the frames occupied by the $n^{th}$ detected object, $T_{1}^{State\;1}$, $T_{2}^{State\;1}$, $T_{1}^{State\;1\&2}$ and $T_{2}^{State\;1\&2}$ are the thresholds to be selected by different road conditions. Therefore, a counted vehicle can be expressed as:

(21) $${Counted}_{n}=\left\{\begin{array}{cc} 0, & \mathrm{if}\;{\mathrm{Detected}}_{n}=\mathrm{noise}\\ 1, & \mathrm{otherwise} \end{array}\right.$$

### 2.4. Evaluation Index

To evaluate the average performance of one method, there are four main metrics that can be used, which are Accuracy [25], Recall, Precision and *F*-measure [46]. Accuracy is used to evaluate the difference between the counted value and the true value, which can be defined as:

(22) $$Accuracy=1-\frac{\left|Counted\;No.-True\;No.\right|}{True\;No.}$$

where TrueNo. is the true number of vehicles and CountedNo. is the counted number.

Recall is a measure of the success of a method in detecting relevant objects from a set, that is, the percentage of relevant detected objects in all relevant objects, while Precision is the percentage of relevant detected objects in all detected objects. *F*-measure is the weighted harmonic average of Recall and Precision, which combines the results of Recall and Precision. The three metrics can be expressed as:

(23) $$Recall=\frac{TP}{TP+FN},\;Precision=\frac{TP}{TP+FP}$$

(24) $$F-measure=2\times \frac{Recall\times Precision}{Recall+Precision}$$

where TP is the number of true positives (the vehicles which were successfully counted), FN is the number of false negatives (the vehicles that should have been counted but were not), and FP is the number of false positives (the false objects which were counted as the vehicles). Using TP, FN and FP, the TrueNo., CountedNo., and Accuracy could also be expressed as:

(25) TrueNo.=TP+FN,CountedNo.=TP+FP

(26) $$Accuracy=1-\frac{\left|FN-FP\right|}{TP+FN}$$

By analyzing the above formulas, it can be found that Accuracy can only evaluate the overall difference between counted values and true values, and cannot reflect the mistakes of treating noise as vehicle or the mistakes of missing detection. Worse, the outcome of Accuracy may approach 1.0 when the numbers of these two mistakes are close to or even equal. Therefore, a comprehensive analysis of Accuracy and *F*-measure can be more scientific to evaluate the effectiveness of a method.

## 3. Experimental Results

**Figure 11:**
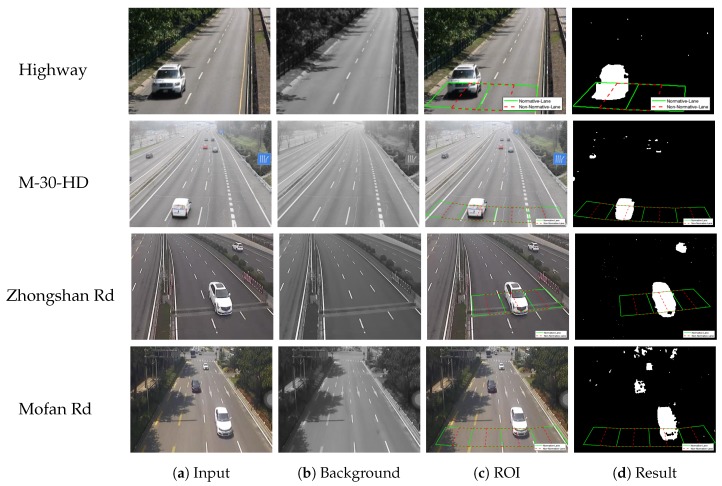
The experimental results.

The results are shown in Figure 11 and Table 6, Table 7, Table 8 and Table 9.

**Figure 12:**
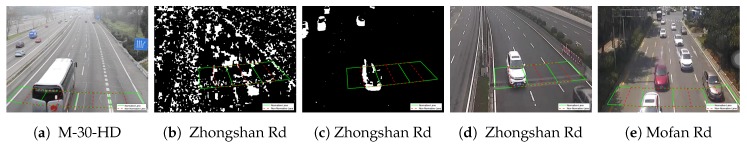
The explanation for errors.

The four metrics for the Highway sequence are both 1.0, which means all the vehicles were successfully counted with our method. For the GRAM-RTM dataset, there was only one redundant error, which was mainly due to the poor shooting angle. Shown in Figure 12a, the large vehicle has been detected in Non-Normative-Lane 1-2 already but was detected again in Normative-Lane 2. However, thanks to the strict restrictions of parameter and state, such situations are not common and only happen on tall and large vehicles.

For the counting results of Zhongshan Rd, the *F*-measure is 0.9937 and the Accuracy is 0.9982. Among the 551 correct targets, there were four redundant errors and three missed errors in the results. Similar to the redundant error in M-30-HD, two redundant errors were due to the poor shooting angle, while the other two were due to the sudden illumination changes, which makes a fault detection, just like Figure 12b shows. For the three missed errors, one of them was due to an incomplete foreground extraction, shown in Figure 12c. For the other two missed vehicles, it was because that they were just driving at the overlap of two adjacent lanes. Just like Figure 12d shows, the missed vehicle was just driving at the overlap of Non-Normative-Lane 1-2 and Normative-Lane 2, which makes the middle proportion values of Non-Normative-Lane 2-3 and Normative-Lane 3 close to zero. In this case, the vehicle was missed.

For the counting results of Mofan Rd, the *F*-measure is 0.9880 and the Accuracy is 0.9983. Among the 578 correct targets, there were six redundant errors and seven missed errors. Similar to the redundant error in M-30-HD, all of them happened on tall and large vehicles. Although this video was shot at a better angle, there is still some tilt distortion when vehicles driving on Normative-Lane 1 (four redundant errors occurred) and Normative-Lane 4 (two redundant errors occurred), especially for the buses. As for the seven missed errors, one of them was due to an incomplete foreground extraction and four missed vehicles were just driving on the overlap of two adjacent lanes. The last two were due to the little space between front and rear vehicles, which happen most often on the relatively congested and slow urban roads, just like Figure 12e shows.

## 4. Discussion

### 4.1. Comparison of Method Accuracy

The comparisons of two benchmark datasets with recent state-of-the-art methods are shown in Table 10. For the CDnet2014 dataset (Highway), the accuracy of the proposed method is 100% with no error in Highway sequence. For the GRAM dataset (M-HD-30), the accuracy of the proposed method is a little lower than [27]. However, according to True (the first column in Table 10), our test sample size is larger, which increases the probability of error.

Moreover, the total *F*-measure of [24,25,26,27] and the proposed method are 0.9348, 0.9807, 0.9171, 0.9898 and 0.9925, respectively, while the total Accuracy are 0.8776, 0.9825, 0.9068, 0.9932 and 0.9993.

### 4.2. Summary of Error Types in Experiments

In Section 3, we provided a detailed analysis of the errors in the experiments. For redundant error, a total of 11 errors could be divided into two categories. The first type is due to the effect of sudden illumination changes in cloudy weather and only two errors occurred, which proves that the proposed real-time background model works well. The second type is due to the large and tall vehicles combining with an oblique shooting angle and nine errors occurred. However, it is due to the inherent defect of the video detection approach. For a traffic surveillance video, the camera will tilt and distort the image more or less, even in vertical aerial shots. In this case, a large and tall vehicle has a high probability of being repeatedly detected. Moreover, when a small vehicle is completely hidden behind a large and tall vehicle, the small vehicle can only be detected and counted when it is partially visible, which may lead to delayed detection and counting, or even missing the vehicle. Although this situation did not appear in the experimental results, it does exist.

For missed error, a total of 10 errors could be divided into three categories. The first type is due to the incomplete foreground extraction and only two errors occurred. The second type is due to the little space between front and rear vehicles and only two errors occurred, which may happen on the relatively congested and slow urban roads. The last one is because of the missed vehicles just driving at the overlap of two adjacent lanes and six errors occurred. However, for a total correct number of 1394, the percentage of the six missed errors was only 0.43%, which indicates that the advantages of this setup far outweigh the disadvantages.

Overall, there are 1394 vehicles that needed to be counted in the experiment. Under the proposed system, 1384 were correctly detected, 11 were incorrectly detected and 10 were missed, achieving an *F*-measure of 0.9925 and an Accuracy of 0.9993.

### 4.3. Detection of Non-Normative Driving

The setting of Normative-Lane and Non-Normative-Lane not only increases the accuracy of vehicle counting but also can detect the non-normative driving, just like Figure 2 shows.

According to Table 11, the percentages of non-normative driving accounted for the total vehicles are relatively lower for the two benchmark datasets on the highway. However, the percentages are high on the expressway and urban road, and even reached 18.90%. In this kind of complex traffic environment, it is necessary to divide the lane into Normative-Lane and Non-Normative-Lane for study, which plays a key role in improving the accuracy of detection and counting.

### 4.4. Robustness to Challenging Detection Environments

We introduced many challenging undesirable factors into our experimental scenes. The adverse weather factors include sudden illumination changes and vehicle shadows, while the complex traffic conditions include a high proportion of large vehicles, high traffic density and serious non-normative driving. Also, poor camera shooting angle was included in our study.

Under the challenge of such adverse influences, the proposed algorithm still achieved a high performance with an *F*-measure of 0.9925 and an Accuracy of 0.9993, which benefits from both the vehicle detection part and the counting part. In the vehicle detection part, the establishment of real-time background and the removal of vehicle shadows have effectively and accurately extracted the foreground. As for the counting, the setting of Normative-Lane and Non-Normative-Lane contributes the most, effectively avoiding multiple and missed counting. Moreover, the strict restrictions of the corresponding parameter and state also improve the accuracy of the algorithm.

## 5. Conclusions

In this paper, we presented a vehicle detection and counting method that can adapt to several challenging detection environments well. The real-time background model was set to resist sudden illumination changes, while the proposed detection algorithm based on motion could remove vehicle shadows successfully. As for counting, the setting of Normative-Lane and Non-Normative-Lane improved the counting accuracy and has realized the function of non-normative driving detection.

Experimental results have shown that the proposed system performs well in challenging detection environments, such as sudden illumination changes and vehicle shadows. Moreover, this system is applicable to both highways and complex urban roads. The proposed algorithm has successfully counted the vehicles with a high performance, for example, the average *F*-measure and Accuracy achieved 0.9928 and 0.9993.

In future works, we intend to optimize the detection algorithms by giving proposed regions of objects to improve the robustness and integrity. Also, deep learning models may be applied in the counting part to reduce the chance of misidentification.

## Figures and Tables

**Table 1:** Comparison of existing state-of-the-art methods.

	Function	Applicable Scenes	Experimental Environments
Bouvié [24]	Detection, counting	Less ideal	Sudden illumination changes and general urban traffic
Quesada [25]	Counting	Less ideal	Sudden illumination changes and general urban traffic
Yang [26]	Detection, counting	Challenging	Sudden illumination change
Abdelwahab [27]	Detection, counting	Ideal	Sudden illumination changes
Proposed Method	Detection, counting and non-normative driving	Challenging and complex	Sudden illumination changes, complex urban traffic and vehicle shadows

**Table 2:** The attributes of the datasets.

Dataset	Road and Environment Properties			Video Properties		
	Location	Lane No.	Weather	Frame No.	Original Size	Adjusted Size
**Highway**	Highway	2	Sunny	1700	320 × 240	320 × 240
**M-30-HD**	Highway	4	Cloudy	9390	1200 × 720	640 × 480
**Zhongshan Rd**	Expressway	3	Cloudy	54000	800 × 600	600 × 450
**Mofan Rd**	Urban road	4	Sunny	54000	800 × 600	600 × 450

**Table 3:** The characteristics and challenges of each road.

Dataset	Scene		Weather		Traffic Conditions			Overall Evaluation
	Camera Shooting Angle	Waving Trees	Sudden Illumination Changes	Vehicle Shadows	Large Vehicle Proportion	Traffic Density	Non- Normative Driving	
**Highway**	Poor	Median	Median	A few	None (0%)	Median	None	**Less ideal**
**M-30-HD**	Not bad	None	A Lot	None	Low (0.85%)	Low	A little	**Not too bad**
**Zhongshan Rd**	Poor	None	A Lot	None	Low (0.91%)	High	Median	**A bit poor**
**Mofan Rd**	Good	A Lot	Median	A Lot	High (8.06%)	High	Much	**Very poor**

**Table 4:** The instruction of ROI.

Lane No.	ROI	Detection Area	Front Line	Back Line	Left Line	Middle Line	Right Line
Normative-Lane 1	${ROI}^{1\&2}$	${Area}^{1\&2}$	${Front}^{1\&2}$	${Back}^{1\&2}$	${Vertical}^{1}$	${Vertical}^{2}$	${Vertical}^{3}$
Non-Normative-Lane 1-2	${ROI}^{2\&3}$	${Area}^{2\&3}$	${Front}^{2\&3}$	${Back}^{2\&3}$	${Vertical}^{2}$	${Vertical}^{3}$	${Vertical}^{4}$
Normative-Lane 2	${ROI}^{3\&4}$	${Area}^{3\&4}$	${Front}^{3\&4}$	${Back}^{3\&4}$	${Vertical}^{3}$	${Vertical}^{4}$	${Vertical}^{5}$
Non-Normative-Lane 2-3	${ROI}^{4\&5}$	${Area}^{4\&5}$	${Front}^{4\&5}$	${Back}^{4\&5}$	${Vertical}^{4}$	${Vertical}^{5}$	${Vertical}^{6}$
Normative-Lane 3	${ROI}^{5\&6}$	${Area}^{5\&6}$	${Front}^{5\&6}$	${Back}^{5\&6}$	${Vertical}^{5}$	${Vertical}^{6}$	${Vertical}^{7}$
Non-Normative-Lane 3-4	${ROI}^{6\&7}$	${Area}^{6\&7}$	${Front}^{6\&7}$	${Back}^{6\&7}$	${Vertical}^{6}$	${Vertical}^{7}$	${Vertical}^{8}$
Normative-Lane 4	${ROI}^{7\&8}$	${Area}^{7\&8}$	${Front}^{7\&8}$	${Back}^{7\&8}$	${Vertical}^{7}$	${Vertical}^{8}$	${Vertical}^{9}$

**Table 5:** Judgement of $State_{k}^{n\&n+1}$.

$State_{k}^{n\&n+1}$	Parameter Restrictions of Occupied Proportion						State Restrictions		
	$Area_{k}^{n\&n+1}$	$Front_{k}^{n\&n+1}$	$Back_{k}^{n\&n+1}$	$Vertical^{n}$	$Vertical^{n+1}$	$Vertical^{n+2}$	$State_{k-1}^{n\&n+1}$	$State_{k}^{n-1\&n}$	$State_{k}^{n+1\&n+2}$
0	$<T^{Area}$	$<T^{Front}$	$<T^{Back}$	$<T^{Left}$	$<T^{Mid}$	$<T^{Right}$	2 or 0	-	-
1	$>T^{Area}$	$>T^{Front}$	$<T^{Back}$	$<T^{Left}$	$>T^{Mid}$	$<T^{Right}$	0 or 1	2 or 0	2 or 0
2	$>T^{Area}$	$<T^{Front}$	$>T^{Back}$	$<T^{Left}$	$>T^{Mid}$	$<T^{Right}$	1 or 2	-	-

Note: $State_{k}^{n\&n+1}$ represents the state of $ROI^{n\&n+1}$ in Frame *k*.

**Table 6:** The counting results of highway.

Lane No.	True	Counted	TP	FN	FP	Recall	Precision	*F*-measure	Accuracy
Normative-Lane 1	17	17	17	0	0	1.0	1.0	1.0	1.0
Non-Normative-Lane 1-2	0	0	0	0	0	1.0	1.0	1.0	1.0
Normative-Lane 2	10	10	10	0	0	1.0	1.0	1.0	1.0
Total	27	27	27	0	0	1.0	1.0	1.0	1.0

**Table 7:** The counting results of M-30-HD.

Lane No.	True	Counted	TP	FN	FP	Recall	Precision	*F*-measure	Accuracy
Normative-Lane 1	66	66	66	0	0	1.0	1.0	1.0	1.0
Non-Normative-Lane 1-2	5	5	5	0	0	1.0	1.0	1.0	1.0
Normative-Lane 2	71	72	71	0	1	1.0	0.9861	0.9930	0.9859
Non-Normative-Lane 2-3	7	7	7	0	0	1.0	1.0	1.0	1.0
Normative-Lane 3	54	54	54	0	0	1.0	1.0	1.0	1.0
Non-Normative-Lane 3-4	0	0	0	0	0	1.0	1.0	1.0	1.0
Normative-Lane 4	31	31	31	0	0	1.0	1.0	1.0	1.0
Total	234	235	234	0	1	1.0	0.9957	0.9979	0.9957

**Table 8:** The counting results of Zhongshan Rd.

Lane No.	True	Counted	TP	FN	FP	Recall	Precision	*F*-measure	Accuracy
Normative-Lane 1	108	107	107	1	0	0.9907	1.0	0.9953	0.9907
Non-Normative-Lane 1-2	45	44	44	1	0	0.9778	1.0	0.9888	0.9778
Normative-Lane 2	214	216	214	0	2	1.0	0.9907	0.9953	0.9907
Non-Normative-Lane 2-3	26	25	25	1	0	0.9615	1.0	0.9804	0.9615
Normative-Lane 3	158	160	158	0	2	1.0	0.9875	0.9937	0.9873
Total	551	552	548	3	4	0.9964	0.9928	0.9937	0.9982

**Table 9:** The counting results of Mofan Rd.

Lane No.	True	Counted	TP	FN	FP	Recall	Precision	*F*-measure	Accuracy
Normative-Lane 1	34	37	33	1	4	0.9706	0.8919	0.9296	0.9118
Non-Normative-Lane 1-2	29	29	29	0	0	1.0	1.0	1.0	1.0
Normative-Lane 2	173	171	171	2	0	0.9884	1.0	0.9942	0.9884
Non-Normative-Lane 2-3	53	50	50	3	0	0.9434	1.0	0.9709	0.9434
Normative-Lane 3	171	170	170	1	0	0.9942	1.0	0.9971	0.9942
Non-Normative-Lane 3-4	28	28	28	0	0	1.0	1.0	1.0	1.0
Normative-Lane 4	94	96	94	0	2	1.0	0.9792	0.9895	0.9787
Total	582	581	575	7	6	0.9880	0.9897	0.9888	0.9983

**Table 10:** The comparison of experimental results with existing state-of-the-art methods.

Method	Highway					M-30-HD				
	True	FN	FP	*F*-measure	Accuracy	True	FN	FP	*F*-measure	Accuracy
Bouvié [24]	N/A	N/A	N/A	N/A	N/A	42	9	0	0.8800	0.7857
Quesada [25]	N/A	N/A	N/A	N/A	N/A	42	3	0	0.9630	0.9286
Yang [26]	16	2	0	0.9412	0.8750	42	5	0	0.9438	0.8810
Abdelwahab [27]	27	0	2	0.9643	0.9259	42	0	0	1.0	1.0
Proposed Method	27	0	0	1.0	1.0	234	0	1	0.9979	0.9957

**Table 11:** Non-normative driving in each scene.

	Total No.	Driving on the Normative-Lane	Driving on the Non-Normative-Lane	Driving on the Non-Normative-Lane (%)
Highway	27	27	0	0%
M-30-HD	234	222	12	5.13%
Zhongshan Rd	551	480	71	12.89%
Mofan Rd	582	472	110	18.90%

## References

[1] Celik T., Kusetogullari H. (2010). Solar-Powered Automated Road Surveillance System for Speed Violation Detection. IEEE Trans. Ind. Electron..

[2] Yang Z., Pun-Cheng L.S.C. (2018). Vehicle detection in intelligent transportation systems and its applications under varying environments: A review. Image Vis. Comput..

[3] Liu Y., Tian B., Chen S., Zhu F., Wang K. A survey of vision-based vehicle detection and tracking techniques in ITS. Proceedings of the 2013 IEEE International Conference on Vehicular Electronics and Safety.

[4] Yang Y., Gao X., Yang G. (2011). Study the method of vehicle license locating based on color segmentation. Procedia Eng..

[5] Haralick R., Shanmugam K., Dinstein I. (1973). Textural features for image classification. IEEE Trans. Syst. Man Cybern..

[6] Matthews N., An P., Charnley D., Harris C. (1996). Vehicle detection and recognition in greyscale imagery. Control Eng. Pract..

[7] Bertozzi M., Broggi A., Castelluccio S. (1997). Real-time oriented system for vehicle detection. J. Syst. Archit..

[8] Teoh S.S., Braunl T. (2012). Symmetry-based monocular vehicle detection system. Mach. Vis. Appl..

[9] Chen D.Y., Lin Y.H., Peng Y.J. (2012). Nighttime brake-light detection by Nakagami imaging. IEEE Trans. Intell. Transp. Syst..

[10] Abolghasemi V., Ahmadyfard A. (2009). An edge-based color-aided method for license plate detection. Image Vis. Comput..

[11] Yang J., Wang Y., Sowmya A., Li Z. Vehicle detection and tracking with low-angle cameras. Proceedings of the 2010 IEEE International Conference on Image Processing.

[12] Lin L., Wu T., Porway J., Xu Z. (2009). A stochastic graph grammar for compositional object representation and recognition. Pattern Recognit..

[13] Krizhevsky A., Sutskever I., Hinton G.E. (2017). ImageNet classification with deep convolutional neural networks. Commun. ACM.

[14] Abdelwahab M.A. (2019). Accurate Vehicle Counting Approach Based on Deep Neural Networks. Proceedings of the 2019 International Conference on Innovative Trends in Computer Engineering (ITCE).

[15] Kim J.B., Kim H.J. (2003). Efficient region-based motion segmentation for a video monitoring system. Pattern Recognit. Lett..

[16] Zhang W., Wu Q.J., Yin H.b. (2010). Moving vehicles detection based on adaptive motion histogram. Digit. Signal Process. Rev. J..

[17] Asaidi H., Aarab A., Bellouki M. (2014). Shadow elimination and vehicles classification approaches in traffic video surveillance context. J. Vis. Lang. Comput..

[18] Shou Y.W., Lin C.T., Yang C.T., Shen T.K. (2010). An efficient and robust moving shadow removal algorithm and its applications in ITS. Eurasip J. Adv. Signal Process..

[19] Zhang X., Gao H., Xue C., Zhao J., Liu Y. (2018). Real-time vehicle detection and tracking using improved histogram of gradient features and Kalman filters. Int. J. Adv. Robot. Syst..

[20] Abdelwahab M.A., Abdelwahab M.M. A Novel Algorithm for Vehicle Detection and Tracking in Airborne Videos. Proceedings of the 2015 IEEE International Symposium on Multimedia (ISM).

[21] Kadikis R., Freivalds K., Verikas A., Vuksanovic B., Zhou J. (2013). Vehicle Classification in Video Using Virtual Detection Lines. Sixth International Conference on Machine Vision (icmv 2013).

[22] Xu H., Zhou W., Zhu J., Huang X., Wang W. (2017). Vehicle counting based on double virtual lines. Signal Image Video Process..

[23] Barcellos P., Bouvie C., Escouto F.L., Scharcanski J. (2015). A novel video based system for detecting and counting vehicles at user-defined virtual loops. Expert Syst. Appl..

[24] Bouvié C., Scharcanski J., Barcellos P., Escouto F.L. Tracking and counting vehicles in traffic video sequences using particle filtering. Proceedings of the 2013 IEEE International Instrumentation and Measurement Technology Conference (I2MTC).

[25] Quesada J., Rodriguez P. Automatic vehicle counting method based on principal component pursuit background modeling. Proceedings of the 2016 IEEE International Conference on Image Processing (ICIP).

[26] Yang H., Qu S. (2018). Real-time vehicle detection and counting in complex traffic scenes using background subtraction model with low-rank decomposition. IET Intell. Transp. Syst..

[27] Abdelwahab M.A. (2019). Fast approach for efficient vehicle counting. Electron. Lett..

[28] The Input of Videos. Extraction Code: wp8v. https://pan.baidu.com/s/1zjp9S1xIUwluQDPgGodSTA.

[29] Change Detection Benchmark Web Site. http://jacarini.dinf.usherbrooke.ca/dataset2014/.

[30] GRAM Road-Traffic Monitoring. http://agamenon.tsc.uah.es/Personales/rlopez/data/rtm/.

[31] Stauffer C., Grimson W.E.L. (2000). Learning patterns of activity using real-time tracking. IEEE Trans. Pattern Anal. Mach. Intell..

[32] Gloyer B., Aghajan H.K., Siu K.Y.S., Kailath T. (1995). Video-based freeway-monitoring system using recursive vehicle tracking. Proc. SPIE.

[33] Stringa E., Regazzoni C.S. (2000). Real-time video-shot detection for scene surveillance applications. IEEE Trans. Image Process..

[34] Wan Q., Wang Y. Background subtraction based on adaptive non-parametric model. Proceedings of the 2008 7th World Congress on Intelligent Control and Automation.

[35] Barnich O., Van Droogenbroeck M. (2011). ViBe: A universal background subtraction algorithm for video sequences. IEEE Trans. Image Process..

[36] Toyama K., Krumm J., Brumitt B., Meyers B. (1999). Wallflower: Principles and practice of background maintenance. Proceedings of the 1999 7th IEEE International Conference on Computer Vision (ICCV’99).

[37] Chung K.L., Lin Y.R., Huang Y.H. (2009). Efficient shadow detection of color aerial images based on successive thresholding scheme. IEEE Trans. Geosci. Remote Sens..

[38] Otsu N. (1979). Threshold selection method from gray-level histograms. IEEE Trans. Syst. Man Cybern..

[39] Cucchiara R., Grana C., Piccardi M., Prati A. (2003). Detecting moving objects, ghosts, and shadows in video streams. IEEE Trans. Pattern Anal. Mach. Intell..

[40] Canny J., Fischler M.A., Firschein O. (1987). A Computational Approach to Edge Detection. Readings in Computer Vision.

[41] Soille P. (2003). Morphological Image Analysis-Principles and Applications.

[42] Lim J.S. (1990). Image enhancement. Two-Dimensional Signal and Image Processing.

[43] Gonzalez R.C., Woods R.E. (2007). Morphological Image Processing. Digital Image Processing.

[44] Roess R.P., Prassas E.S., McShane W.R. (2011). Road user and vehicle characterstics. Traffic Engineering.

[45] (2004). A Policy on Geometric Design of Highways and Streets.

[46] Wang Y., Jodoin P.M., Porikli F., Konrad J., Benezeth Y., Ishwar P. CDnet 2014: An Expanded Change Detection Benchmark Dataset. Proceedings of the 2014 IEEE Conference on Computer Vision and Pattern Recognition Workshops.

